# An update on the tools for creating transgenic animal models of human diseases – focus on atherosclerosis

**DOI:** 10.1590/1414-431X20198108

**Published:** 2019-04-25

**Authors:** A.S. Volobueva, A.N. Orekhov, A.V. Deykin

**Affiliations:** 1 Institute of Gene Biology, Russian Academy of Sciences, Moscow, Russia; 2 Laboratory of Gene Therapy, Biocad Biotechnology Company, Strelnya, Russia; 3 Laboratory of Angiopathology, Institute of General Pathology and Pathophysiology, Moscow, Russia; 4 Institute for Atherosclerosis Research, Skolkovo Innovative Center, Moscow, Russia

**Keywords:** Animal models, Gene editing, Atherosclerosis

## Abstract

Animal models of diseases are invaluable tools of modern medicine. More than forty years have passed since the first successful experiments and the spectrum of available models, as well as the list of methods for creating them, have expanded dramatically. The major step forward in creating specific disease models was the development of gene editing techniques, which allowed for targeted modification of the animal's genome. In this review, we discuss the available tools for creating transgenic animal models, such as transgenesis methods, recombinases, and nucleases, including zinc finger nuclease (ZFN), transcription activator-like effector nuclease (TALEN), and CRISPR/Cas9 systems. We then focus specifically on the models of atherosclerosis, especially mouse models that greatly contributed to improving our understanding of the disease pathogenesis and we outline their characteristics and limitations.

## Introduction

Model organisms are widely used in biomedicine for studying the pathophysiology of human diseases and developing novel therapeutic and diagnostic methods. Naturally occurring animal models of human genetic diseases, like Watanabe rabbits with heritable hypercholesterolemia, are rare, probably, because of poor survival of such animals. The era of transgenic animals began in 1974 when Rudolf Jaenisch managed to insert DNA into mice embryos and obtained the first genetically modified animal (1). Since then, plenty of methods have been applied to establish somatic and germline animal models of different human diseases, mostly using mouse models (2). Among the most used methods are oocyte pronuclear DNA microinjection, intracytoplasmic sperm injection, stem cells modifications, and somatic cell nuclear transfer. The basic strategies for creating disease models are artificial addition of a gene into the animal cells (knock-in), switching off a certain gene (knock-out), and conditional gene modification in response to certain stimuli or in a specific tissue environment. These approaches are based on homologous recombination, which rarely happens in nature. Viral vectors were very helpful for gene transfer, but in most cases caused uncontrolled and often random transgene integration, which affected reproducibility and reliability of resulting models. Moreover, uncontrolled gene expression could affect multiple pathways in the animal model organism making it difficult to replicate specific disease conditions. Therefore, the development of site-specific gene integration was a crucial step forward in creating genetically modified animals with accurate and identical genotypes. Novel genetic tools allowing for targeted changes via homologous recombination with higher frequency have been designed, including zinc finger nucleases (ZFN), transcription activator-like effector nucleases (TALEN), and clustered regularly interspaced short palindromic repeats (CRISPR)/Cas9. In this review, we will summarize the existing approaches to genetically modified animal development with special focus on transgenic animal models of atherosclerosis.

## Techniques of transgenesis

Generating transgenic mice using oocyte pronuclear DNA microinjection includes five basic steps: purification of transgenic construct, harvesting donor zygotes, microinjection of transgenic construct, implantation of microinjected zygotes into pseudo-pregnant recipient mice, and genotyping of transgene expression in founder mice (3). Different vectors for DNA delivery have been explored: episomal and viral vectors, as well as artificial chromosomes allowing for insertion of large fragments.

Intracytoplasmic sperm injection (ICSI) has higher efficiency of transfer of foreign DNA in the host genome compared to pronuclear injection. In this method, animal spermatozoa are demembranated either by freeze-thawing or by treatment with a detergent and incubated with linear, double stranded transgenic DNA. This sperm-DNA complex is injected into mature metaphase II oocytes by ICSI, allowing the transgene to be incorporated into the embryonic genome via the DNA repair mechanism (4,5).

Embryonic stem (ES) cells can also be used for transgenesis. The exogenous DNA is introduced into stem cells, following cell propagation and selection steps. After that, the genetically modified ES cells are introduced into the embryo, for instance, by injecting them into a blastocyst using micromanipulators or by aggregating them with eight-cell-stage embryos. These methods have been used for creating mouse models (6).

Somatic cell nuclear transfer (SCNT) remains by far the most popular method for production of large transgenic animals. SCNT involves generation of modified somatic cells (typically fibroblasts), which are then fused with the enucleated oocyte to restart the embryo development. The genetically modified embryos are then transferred into the oviducts of recipient animals (7).

## Molecular tools for gene editing

Recombinases, nucleases, and accessory proteins are natural or artificially created tools for gene editing that are used for developing powerful methods of genetic engineering. We will briefly describe the most commonly used gene editing tools below (Table 1).

**Table 1.** Brief characteristics of available molecular gene editing tools.

Tool	Mechanism	Advantages	Limitations
**Recombinases**			
Cre-LoxP, Flp-FRT, Nigri/nox, Panto/pox and others	Induce recombination between target sites	Conditional gene modification possible	Difficult to find highly specific promoters, costly, and time consuming
**Nucleases**			
ZFN	Recognize a specific site on DNA through zinc finger domains and introduce a double-stranded break	Gene targeting with good efficacy	Off-target activity, technical difficulty to create ZFN modules, difficulty to replace large fragments of DNA, high cost
TALENs	Cleave DNA between TALEN binding sites	A simpler and faster design compared to ZFN facilitated by the creation of “TALEN library”	Off-target activity, relatively large size complicates the delivery
CRISPR/Cas9	Cas9 is guided by RNA and cleaves DNA at designed sites	Ease of use: can be adapted to target any DNA site by changing the guide RNA	Relatively large size of the protein complex, off-target cleavage

ZFN: zinc finger nucleases; TALENs: transcriptional activator-like effector nucleases; CRISPR/Cas9: clustered regularly interspaced short palindromic repeat/CRISPR-associated protein 9.

### Recombinases

Recombinases are enzymes allowing for site-specific mutagenesis. They recognize their target sites and induce recombination between them, thereby excising DNA fragments located between them. Depending on the orientation of the binding sites, recombinases can cause deletions, insertions, translocations, and inversions. One of the major advantages of recombinases is that they allow for conditional gene modification, which is of special importance when mutations in a target gene are lethal during embryogenesis. Using tissue-specific or inducible promotors, researchers can choose the preferential time-point and organ for genetic modification to occur. However, finding a highly specific promotor is challenging and not always possible, and much effort is needed to create an optimal disease model using this method. Among the most known recombinase systems are Cre-LoxP, which was introduced in 1980, and Flp-FRT. Cre-LoxP has been applied for generation of various mouse models of human cancers (8). Recently, novel recombinase systems were discovered, such as Nigri/nox and Panto/pox (9). Among the limitations of this method is the fact that it remains rather costly and time consuming.

### Nucleases

Endonucleases induce double-stranded breaks (DSB) in the DNA molecule that can later be repaired by the cellular DNA repair systems, via either blunt-end, non-homologous end-joining (NHEJ) or by homology-directed repair (HDR). In the case of NHEJ, a proportion of DSBs within the cellular population will be misrepaired leading to heterogeneous genetic insertions or deletions at the target site. By contrast, HDR enables precise insertion of a transgene into the targeted region if the donor DNA template contains the desired transgene flanked by sequences that are homologous to the regions either side of the cleavage site and is co-delivered into the cell along with the nuclease. Genome editing approach using optimized endonuclease systems, such as ZFN, TALEN, and CRISPR/Cas9, are successfully used for generation of both knock-out and knock-in cultured cells and animal strains.

*ZFNs*. ZFNs are fusion proteins that contain a modular array of Cys2-His2 DNA-binding zinc finger domains linked to the endonuclease domain of FokI bacterial restriction enzyme. The zinc finger is one of the most common DNA binding motifs, and one finger interacts with a triplet of nucleotides. To create a ZFN-based gene editing system targeting a particular gene, a pair of ZFN modules needs to be designed: one targeting the forward and one the reverse DNA strand flanking the target sequence. Binding on either side of the site and dimerization of the pair of FokI causes cleavage of the DNA at the specific site with 5′ overhang sequences (7,10). These breaks are normally repaired by NHEJ, resulting in the introduction of the desired sequence in the DNA, although non-specific misrepair is also possible.

ZFNs have been successfully used to generate some animal models of neurological disorders. At SAGE Labs (USA), two first transgenic rat models of autism spectrum disorder (ASD) (syndromic and nonsyndromic) were engineered using ZFNs to knock-out the gene of Fragile X mental retardation protein (*FMRP*) and Neuroligin3 gene (*NLGN3*). According to the results of rapid juvenile test battery, modified rats exhibited abnormalities in ASD-relevant phenotypes, including juvenile play, perseverative behaviors, and sensorimotor gating (11). This work was of special importance, since rat models are preferred to mice in neuroscience studies (12). Neural circuitry in rats is more similar to that in humans. Moreover, rats are characterized by stable behavioral performance and larger brain and cerebral spinal fluid volumes, and are more suitable to surgical manipulations (13).

*TALEN*. TALEN proteins are secreted by *Xanthomonas* bacteria to bind host plant DNA and modify gene expression in favor of bacterial growth. TALE repeats consist of 33–35 amino acids that recognize each of the four possible DNA base pairs by two adjacent hypervariable amino acid residues called repeat variable diresidues (RVDs). Natural bacterial TALE repeats are assembled in arrays of 10 to 30 repeats, with RVDs at positions 12 and 13. These two amino acid residues therefore determine the specific binding to one target nucleotide in the DNA. For gene editing purposes, similar to ZFNs, TALE repeats are fused to the FokI endonuclease domain creating TALEN (7).

TALEN technology has been applied for modelling of epileptic encephalopathy in mice by introduction a missense mutation p.Asn1768Asp in *Scn8a* gene (sodium channel Nav1.6). This mutation exhibits a dominant gain of function due to impaired channel inactivation. Researchers generated several TALEN constructs and targeting template of 4 kb genomic DNA to maximize the yield of targeted alleles. Then, two rounds of injection of both mRNA of TALEN and circular targeting template were made into pronuclei of fertilized mouse oocytes. The yield of live-born animals and mice carrying correctly targeted alleles was 19 and 7%, respectively. The desired p.Asn1768Asp mutation could be transmitted through the germlines of the two founder mice to 50% of heterozygous offspring who exhibited behavioral abnormalities, seizures, and sudden unexpected death in epilepsy (14).

TALEN system was used to modify cynomolgus monkey genome in order to establish monkey model of Rett syndrome, a monogenic human neurodevelopmental disorder caused by loss of function mutations in the *MECP2* gene. To achieve that goal, mature oocytes were fertilized via ICSI procedure, and TALEN coding plasmids were injected into the cytoplasm. The specific disease phenotype was confirmed by the results of behavioral analyses, brain MRI examination, and blood transcriptome profiling. Off-target mutations on all the 41 potential off-target sites from the four new monkeys were not detected (15). Noteworthy, non-human primates may serve as a better model for studying neurological disorders than rats or mice.

Another example of successful implication of TALEN is the zebrafish knock-out of galactose-1-phosphate uridylyltransferase (GALT) enzyme gene (16). TALEN constructs mRNA were injected in wild type one-cell embryos, the fish were raised to adulthood, and outcrossed to generate heterozygotes in the offspring, which were further incrossed in order to generate homozygous, GALT knock-out zebrafish line. This line phenotypically corresponded to a human disease condition with severely impaired GALT activity, decreased motor function, and decreased fertility.

*CRISPR/Cas*. Techniques using CRISPR/Cas pathway provided a new powerful approach for establishing model organisms. CRISPR/Cas9 system functions as an analog of immune system in prokaryotes protecting them from invading viruses. The original system requires about 9 proteins and 2 RNAs: CRISPR RNA (crRNA) and transactivating CRISPR RNA (tracrRNA) that form a complex for directing the Cas9 nuclease. Three types of CRISPR/Cas9 are known. One of them, type II, was optimized for the purposes of genetic engineering, and currently only two components are required for its function: Cas9 protein that enzymatically cuts DNA creating DSB and a single guide RNA (sgRNA) that determines the target DNA sequence for Cas9. Cas9 contains two conserved nuclease domains, HNH and RuvC, that cleave the target DNA strand complementary and non-complementary to the sgRNA, respectively. sgRNA is composed of ‘scaffold’ RNA and ‘spacer’ or ‘targeting’ RNA, which hybridizes a 20-base pair DNA sequence present immediately upstream of an NGG DNA motif (protospacer-associated motif, or PAM). Therefore, the presence of PAM and unique target sequence are prerequisites for proper action of Cas9 on the DNA molecule (17,18). Nevertheless, the guide sequence can tolerate certain mismatches to the DNA target depending on quantity, position, and base identity of mismatches, therefore allowing for undesired off-target mutagenesis. Since the first experiments, some advances in CRISPR/Cas9 engineering have been made. Some modern CRISPR/Cas9 design tools are described elsewhere (19). Different approaches to decrease the off-target activity without compromising the on-target activity were suggested (16). It was noticed that both shortening of sgRNA and adding two guanine nucleotides to its 5′ end, as well as titrating the amount of Cas9 and sgRNA, were not sufficient. Two alternative approaches gave more promising results: the use of Cas9 nickase (Cas9n) or fCas9. Wild-type Cas9 is converted to Cas9n by mutations of the catalytic residues D10A in RuvC and H840A in HNH domains. Double strand breaks (DSBs) can be induced only when two complexes of Cas9n-gRNA bind near a genomic locus on the opposite strands. The advantage of Cas9n is that in case of off-target activity, each Cas9n-sgRNA generates only single strand breaks that are corrected via excision repair mechanisms: damaged nucleotides are replaced using the intact DNA strand (20). It was shown that application of two Cas9n mutant molecules with a pair of sgRNA facilitated high efficiency homology directed repair, NHEJ-mediated DNA insertion, and genomic microdeletions both in cell culture and mouse zygote (21). fCas9 is a fusion protein comprising a catalytically inactive mutant form of Cas9 (“dead” Cas9), which is only able to bind DNA, and the FokI DNA nuclease that induces DSBs upon delivery with two sgRNAs. It is expected to have less of off-target specificity than Cas9 because the distance between the two binding sgRNAs required for DSBs is smaller in fCas9 (14–17 bp) than in Cas9n (4–20 bp). Activity of fCas9 was proven in animals; for instance, mice bearing mutation on the *Bcr* locus were generated by microinjection of fCas9 and gRNAs into fertilized eggs (22).

CRISPR/Cas9 system has been successfully applied to generate various somatic and germline mouse models of cancer reviewed by Mou et al. (23). Complement protein C3 knock-out piglets were generated using modification of fetal porcine fibroblasts via nucleofection with CRISPR/Cas9 coding plasmid followed by SCNT. Out of 300 transferred reconstructed oocytes, 19 alive piglets with C3 knock-out were obtained (24). Pigs are valuable model animals for preclinical research because of their anatomical and physiological similarities with humans.

## Animal models of atherosclerosis

Pathogenesis of atherosclerosis is quite complex and includes several processes. Endothelial dysfunction and increased permeability, combined with altered lipoprotein profile and the presence of modified low-density lipoprotein (LDL), lead to accumulation of cholesterol in the subendothelial space of blood vessel walls. This process is associated with local inflammation, and expression of chemotactic proteins enhance recruitment of the immune cells. Macrophages engulf atherogenic LDL particles and accumulate lipids intracellularly, becoming foam cells that contribute to the formation of atherosclerotic plaque. Increased migration and phenotypic alteration of vascular smooth muscle cells in the arterial wall leads to increased secretion of extracellular matrix proteins. At the later stages, atherosclerotic plaque acquires a fibrous cap that separates it from the blood stream and has protective functions. Plaque destabilization and rupture can cause thrombus formation, which can be followed by thromboembolia with fatal consequences (25). Among risk factors promoting atherosclerotic plaque formation are hyperlipidemia, hyperhomocysteinemia, hyperfibrinogenemia, hypertension, endothelial dysfunction, smoking, male gender, and diabetes (26).

Traditionally, animal models of atherosclerosis with unaltered genotype were obtained by implementing a cholesterol-rich diet. Mice, rabbits, pigs, and non-human primates were the most frequently used species for creating such models. Although numerous genetic variations in lipid metabolism genes are known to be associated with atherosclerosis (26), three main molecular targets were chosen to develop genetically modified animal models: LDL receptor (LDLr), apolipoprotein E, and proprotein convertase subtilisin/kexin type 9 (PCSK9). LDLr binds LDL particles, while apolipoprotein E acts as a ligand for receptors that clear chylomicrons and very low-density lipoprotein (VLDL) and participate in hepatic uptake of lipoprotein particles. Dysfunction of LDLr and ApoE is manifested by increased plasma levels of total cholesterol. Mutations in the *PCSK9* gene are associated with autosomal dominant hypercholesterolemia. PCSK9 directly interacts with the hepatic LDLr and enhances its degradation by targeting it for destruction in the lysosomes (27). Apolipoprotein E-deficient (ApoE^-/-^), LDL-receptor (LDLr) knock-out, ApoE/LDLr double-knock-out, ApoE3-Leiden, and PCSK9-AAV mice are all valuable tools in atherosclerosis research.

Historically, ApoE knock-out mice were the first animal model of atherosclerosis established using gene technology methods. This model was a great achievement of the time that not only provided a useful tool for studying atherosclerosis, but also opened the door for the development of other genetically modified animal models of the disease. To establish ApoE knock-out mice lines, Piedrahita with co-authors disrupted *ApoE* gene in mouse ES cells using electroporation with plasmids of the replacement type to induce HR, after which, modified ES cells were injected into mouse blastocysts. Resulting chimeras were crossed to obtain homozygous lines (28,29). Comparable ApoE-deficient mice were created in parallel by Plump et al. (30). ApoE-deficient mice developed hypercholesterolemia and atherosclerotic lesions, which allowed using them as a small animal model of the disease. Later, another double knock-out line was produced by Bonthu et al. by mating previously generated homozygous ApoE knock-outs and LDLr knock-out lines (31).

Shortly after generation of ApoE knock-outs, another approach to generating dyslipidemic mice was taken by creating animals that bear a human gene variant associated with familial dysbetalipoproteinemia. APOE*3-Leiden dominant mutation was present in a Dutch family. ApoE3-Leiden mice were designed by Van den Maagdenberg and co-authors using microinjection of cosmid carrying the desired mutated DNA fragment into male pronuclei of fertilized mouse eggs taken from super-ovulated females (32). ApoE3-Leiden mice have a human-like lipoprotein profile (an increase in VLDL/LDL particles) and functional ApoE. They exhibit signs of late atherosclerosis, including the presence of foam cells, a large necrotic core, intra-plaque neovascularization, calcification, and cholesterol clefts.

To generate PCSK9^mut^ mice, adeno-associated virus carrying gain-of-function mutant PCSK9^DY^ was intravenously injected into mice. PCSK9^mut^-mice showed hyperlipidemia and a build-up of lesions similar to other models and were easier to create. Noteworthy, combined PCSK9^DY^ expression and ApoE deficiency caused more pronounced hyperlipidemia than each of the single modifications (33,34).

Despite their wide use, the described rodent models have their limitations. Large differences in lifespan, metabolism, and physiology make it difficult to translate the findings obtained in mouse models into clinical practice. For instance, in ApoE^-/-^ mice, like in ApoE/LDLr double knock-outs, VLDL is the most abundant lipoprotein instead of LDL. The major limitation of ApoE^-/-^ mice is the rarity of plaque rupture and thrombosis. To model the specific situation of plaque rupture, a perivascular collar or cuff was used. Mice carrying mutation in the gene coding for fibrillin-1, a structural component of the extracellular matrix microfibrils, were generated by crossing mice from the already established ApoE^-/-^ and C1039G lines. In the blood vessel wall, microfibrils form the periphery of the elastic fiber, acting as a scaffold for elastin deposition. ApoE^-/-^Fbn1^C1039G+/-^ mice develop larger and highly unstable plaques with a large necrotic core and strongly diminished collagen content. Upon Western-type diet, these mice acquired fibrin-rich mural thrombi in brachiocephalic, carotid, and coronary arteries, and ascending aortas (35).

Larger animal models of atherosclerosis have been developed, including atherosclerotic rabbits and pigs. Like humans, rabbits are very susceptible to diet-induced atherosclerosis. Unmodified Watanabe heritable hyperlipidemic rabbits with mutant LDLr and New Zealand rabbits fed a cholesterol-rich diet develop all signs of atherosclerosis. Recently, ApoE^-/-^ rabbits were obtained by two research groups. One group used ZFNs coding plasmids for pronuclear microinjection of fertilized oocytes from New Zealand female rabbits (36). The other group microinjected Cas9 mRNA and sgRNA into the cytoplasm of pronuclear stage rabbit embryos (37). When animals were fed with a high fat diet, both lines developed hyperlipidemia and lesions, mainly composed of macrophage-derived foam cells.

Several pig models with LDLr knock-out also exist. Authors transfected conventional targeting vector for porcine *LDLR* gene into swine fibroblasts and then performed somatic cell nuclear transfer (38). Animals were fed with a fat diet and were subjected to balloon injury to accelerate the atherosclerotic process. Advanced coronary atherosclerotic lesions with lipid pools were observed eight weeks after balloon injury with fibrous components becoming visible at 12 weeks.

Today, a variety of animal models of atherosclerosis is available that allow studying different aspects of the disease, with each model having its strengths and limitations (Table 2). These models have already contributed greatly to our understanding of the disease pathogenesis. However, despite this improved understanding, relatively little progress has been achieved in curative atherosclerosis therapy and its effective prevention. Available therapies are mostly symptomatic in nature and are of little help for reducing atherosclerotic plaques that are already formed. One of the important future directions in atherosclerosis research is identifying possible novel therapies, and animal models will be an invaluable tool in that search.

**Table 2.** Genetically engineered animal models of atherosclerosis.

Model	Method of creation	Characteristics	References
ApoE^-/-^ mice	Targeting the *ApoE* gene in embryonic stem cells by homologous recombination	No ApoE present in the blood, hypercholesterolemia, atherosclerotic lesions	[Bibr B28] 28,30
ApoE/LDLr double-knock-out mice	Crossing of homozygous ApoE knock-out and LDLr knock-out lines	Demonstrate vascular remodeling and intimal thickening, accelerated atherosclerosis development compared to ApoE single knock-out	31
ApoE*3-Leiden mice	Injection of the DNA fragment into fertilized eggs	Elevated plasma cholesterol and triglycerides, diet-induced hypercholesterolemia comparable to that in affected humans	32
PCSK9 D374Y gain-of-function mutant mice	Injection of adeno-associated virus (AAV) vector into ApoE^-/-^ mice	Greatly increased atherosclerotic lesions compared to ApoE knock-out	[Bibr B33] 33,34
ApoE^-/-^ rabbits	ZFN, Cas9 techniques	Increased cholesterol and triglycerides levels, diet-induced hyperlipidemia, atherosclerotic lesions	[Bibr B36] 36,37
LDLr^-/-^ pigs	Deletion of LDLr in cultured fibroblasts and cloning knock-out embryos	Rapid development of balloon injury-induced coronary atherosclerosis	38

ApoE^-/-^: apolipoprotein E-deficient; LDLr: low-density lipoprotein receptor; ZFN: zinc finger nucleases; PCSK9: proprotein convertase subtilisin/kexin type 9.

## Conclusion

Development of novel gene editing techniques had a great beneficial effect on transgenic animal model creation. Nucleases allow for generating larger animal models, which resemble human pathology more closely. Combining different tools, such as CRISPR/Cas9, RNAi, and Cre-loxP allows controlling multiple genetic events independently. Optimization of these systems will continue, and they will be adapted to create animal models that are more precise for studying human diseases.
